# Cryo-electron microscopy structure of a human PRMT5:MEP50 complex

**DOI:** 10.1371/journal.pone.0193205

**Published:** 2018-03-08

**Authors:** David E. Timm, Valorie Bowman, Russell Madsen, Charles Rauch

**Affiliations:** 1 Structural Biology, Discovery Chemistry Research and Technologies, Eli Lilly and Company, Lilly Corporate Center, Indianapolis, Indiana, United States of America; 2 Department of Biological Sciences, Purdue University, West Lafayette, Indiana, United States of America; 3 Structural Biology, Discovery Chemistry Research and Technologies, Eli Lilly and Company, Lilly Biotechnology Center, San Diego, California, United States of America; University of Bern, SWITZERLAND

## Abstract

Protein arginine methyl transferase 5 (PRMT5) is a signaling protein and histone modifying enzyme that is important in many cellular processes, including regulation of eukaryotic gene transcription. Reported here is a 3.7 Å structure of PRMT5, solved in complex with regulatory binding subunit MEP50 (methylosome associated protein 50, WDR77, p44), by single particle (SP) cryo-Electron Microscopy (cryo-EM) using micrographs of particles that are visibly crowded and aggregated. Despite suboptimal micrograph appearance, this cryo-EM structure is in good agreement with previously reported crystal structures of the complex, which revealed a 450 kDa hetero-octameric assembly having internal D2 symmetry. The catalytic PRMT5 subunits form a core tetramer and the MEP50 subunits are arranged peripherally in complex with the PRMT5 N-terminal domain. The cryo-EM reconstruction shows good side chain definition and shows a well-resolved peak for a bound dehydrosinefungin inhibitor molecule. These results demonstrate the applicability of cryo-EM in determining structures of human protein complexes of biomedical significance and suggests cryo-EM could be further utilized to understand PRMT5 interactions with other biologically important binding proteins and ligands.

## Introduction

**Fig 1:**
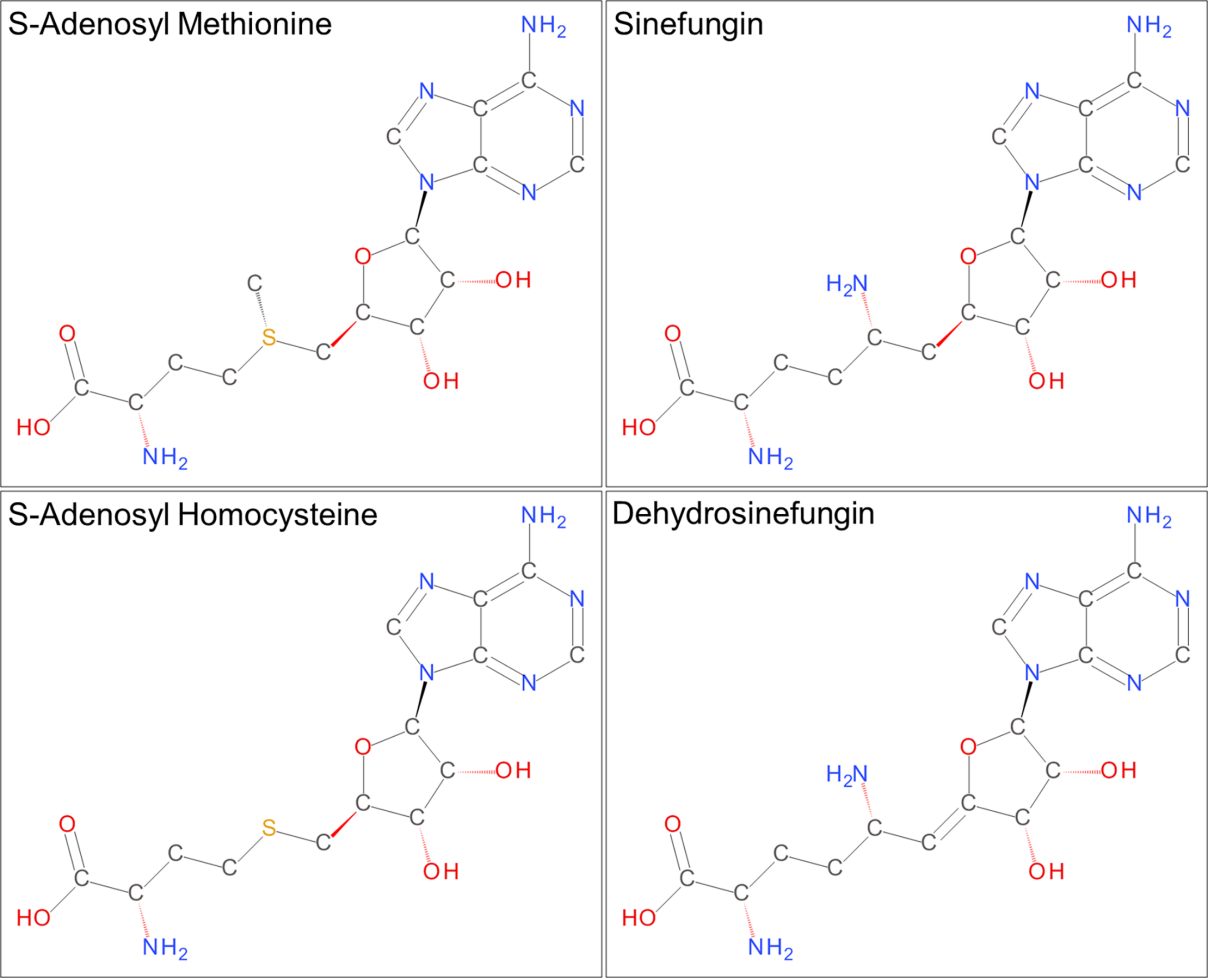
Chemical structures of SAM, SAH, sinefungin and dehydrosinefungin. The amino acid moieties of sinefungin and dehydrosinefungin are joined to the ribose ring through a saturated single bond and a desaturated double bond, respectively.

PRMT5 (capsuleen, Dart5, Hsl7, Jbp1, Skb1) is a key arginine methyl transferase that is involved in a plethora of important cellular functions and clinical disease states. This enzyme catalyzes the non-processive transfer of a methyl group from a S-adenosyl methionine co-factor (SAM, Fig 1) to the ω-nitrogen atoms in the Arg guanidinium group to generate S-adenosyl homocysteine (SAH, Fig 1) and monomethyl Arg and symmetric dimethyl Arg products [1]. Vertebrate PRMT5 is almost always found bound to the adapter protein MEP50 [2], which has been shown to enhance methyl transferase activity for substrates like histones H2A, H3 and H4 by a mechanism that likely involves enhanced substrate binding and recognition via MEP50 interactions with substrates [3]. Histone methylation by PRMT5 is critical to epigenetic regulation of gene transcription and modulates expression of gene targets such as cyclin E1 [4], the Rb tumor suppressor [5] and ribosomal proteins [6]. PRMT5 also impacts gene expression by methylating and thus regulating the activity of important transcription factors such as E2F-1 [7], NF-κB [8] and the p53 tumor suppressor [9]. Additional important proliferative signals are influenced by PRMT5 methylation of the EGF receptor [10] and RAF [11] within the RAS-ERK pathway downstream of the EGF receptor.

The cellular functions of PRMT5 are closely tied to interactions with MEP50 and numerous other PRMT5 adapter proteins [12]. By influencing the sub-cellular distribution of PRMT5 and interactions with specific substrates, PRMT5 association with adapter proteins is closely tied to transcriptional regulation, RNA processing [13], chromatin remodeling [14, 15] and other biological roles [12]. While MEP50 forms a stable, heterooctameric complex [16, 17] with PRMT5 (4 [PRMT5:MEP50]), PRMT5 also associates with pICln (nucleotide sensitive chloride channel) to form a complex known as the methylosome that targets cytoplasmic spliceosomal proteins for PRMT5 methylation required for RNA splicing [2]. Cytosolic PRMT5 association with RioK1 (RIO kinase 1; [18]) mediates association with the RNA-binding protein nucleolin in a mutually exclusive manner with MEP50, while association with CoPR5 (cooperator of PRMT5) is believed to recruit PRMT5 to the nucleus [19].

PRMT5 dysfunction and/or dysregulation is of particular interest in cancer biology [20] and PRMT5 inhibitors have been researched as potential chemotherapeutics [13, 21, 22]. PRMT5 amplifications, deletions and mutations occur with significant frequency in many cancer types [12], including uterine, head and neck, bladder, ovarian, gastric, and lung. PRMT5 overexpression results in tumors in nude mice [23], increased cellular proliferation and anchorage-independent colony formation [23, 24]. High PRMT5 expression is also associated with poor clinical prognoses [21, 25]. Some of the earliest identified inhibitors of PRMT5, including sinefungin and dehydrosinefungin [26], are natural products that share high chemical similarity with SAM and SAH (Fig 1). Given the complexity of PRMT5 function it may be challenging to identify safe PRMT5 inhibitors that target a specific proliferative mechanism.

Rapid, exponential growth in single particle cryo-EM derived structures has been underway for the last several years [27]. Notable milestone achievements in the field are well-documented and have included high resolution breakthroughs in the low 2 Å range and beyond [28–30], dramatic reductions in the molecular weight limit of samples suitable for study [31] and applications to targets that have proven intractable to X-Ray crystallography [32–34]. The Electron Microscopy Data Bank [35] currently contains over 620 maps solved by SP cryo-EM at resolutions better than 4 Å. However, maps from human sources are less common (~15%), and fewer still are maps for lower molecular weight human enzymes typical of historic structure-based drug design targets. The PRMT5:MEP50 complex was studied as a human drug discovery target of moderate molecular weight (~450 kDa) to further evaluate the potential for solving structures of targets of pharmaceutical interest using SP cryo-EM. The results obtained revealed good levels of structural detail for PRMT5:MEP50 that were consistent with crystallographic results [16], and clearly indicated the position and orientation of a known SAM competitive inhibitor within the complex.

## Materials and methods

### Protein sample

The PRMT5:MEP50 complex was prepared as previously described [16]. Briefly, full-length human PRMT5 (residues 1–637, NP_006100) and MEP50 (residues 2–342, NP_077007) were co-infected and co-expressed in Sf9 cells (Invitrogen/Thermo Fisher). Following lysis and removal of debris the complex was purified at 4° C. by FLAG-affinity chromatography (Sigma; A2220; F3290), followed by gel filtration on a 16/60 S300 column (GE Life Sciences) in 10 mM Hepes, pH 7.5, 0.15 M NaCl, 10% (vol/vol) glycerol, 2 mM DTT (see supplementary information S1 Fig). The sample was concentrated to 11.5 mg/mL and stored at -80° C. Substrate H4 peptide [16] is not included in the sample.

### Negatively stained grid preparation

Negatively stained grids were prepared using uranyl formate as previously described [36], with 3.5 μL of the protein sample described above (diluted between 1:20 and 1:50 vol/vol with a buffer containing 10 mM Hepes, pH7.5, and 150 mM NaCl) spotted onto a 400 mesh copper grid coated with ultrathin carbon (Electron Microscopy Sciences, CF400-CU-UL) that had been glow discharged for 40 seconds using an Emitech K950 glow-discharge unit.

### Cryo-EM grid preparation

Cryo-EM grids were prepared by spotting 3.5 μL of the protein sample (diluted 1:30 vol/vol with a buffer containing 10 mM Hepes, pH7.5, 150 mM NaCl and 3.33 mM dehydrosinefungin) onto holey carbon 400 mesh Cu grids (Quantifoil R2/1) that had been glow discharged as described above. Sample was incubated on the grids for 30 seconds at 80% humidity in a Gatan Cryoplunge 3 device; grids were sandwich blotted for 1.8 seconds, plunged in liquid ethane and transferred to liquid nitrogen for storage.

### Imaging

Negatively stained grids were imaged with 1 second exposures at an actual magnification of 50,607x using a Phillips CM200 microscope operating at 200 kV and equipped with a Gatan US4000 CCD camera under the control of Gatan DIGITAL MICROGRAPH software at a defocus of 1 μm.

The 3D reconstruction utilized 193 cryo-EM movie stacks collected using the LEGINON/APPION interface [37] to automate operation of a FEI Titan Krios microscope at 300 kV with an actual magnification of 50,000x and a pixel size of 1 Å^2^. Each movie stack contained 40 frames collected with an exposure time of 0.2 seconds/frame and a dose rate of 1.6 electrons/pixel/second using a Gatan K2 Summit direct electron detector operated in counting mode. Defocus values were randomly set to between 0.85 and 2.5 μm.

### Image processing and 3D reconstruction

Movie frames were summed and dose-weighted using MOTIONCOR2 [38]. CTF estimates and defocus values ranging from 0.7 to 5.1 μm were calculated from the summed images without dose-weighting using CTFFIND4 [39]. Particles extracted from summed, dose-weighted images were utilized in subsequent steps. RELION1.4 [40, 41] was used for particle picking, extraction, classification and refinement. In an attempt to deal with particle crowding and aggregation, automated picking was carried out using a relatively small particle mask diameter (164 Å) and minimum inter-particle distance (100 Å) to generate a set of 113,167 particles using six 2D class average references derived from initial manual particle picking. Two rounds of 2D class averaging were used to select a sub-set of 89,837 particles used for 3D classification, and 26,553 particles from a single 3D class exhibiting higher resolution features were used in 3D auto-refinement and post-processing. Particles excluded following 2D and 3D classifications included classes of ice or ethane contaminants, carbon edges, mis-centered and/or aggregated particles, bad pixel artifacts, and featureless, low contrast classes. No classes were identified for distinct conformational states or sub-stoichiometric assemblies of PRMT5 or MEP50. The final resolution is estimated as 3.7 Å based on FSC = 0.143 criterion. The map was sharpened using RELION1.4 using the auto-masking option (initial binarization threshold of 0.014, binary map extended by 3 pixels, soft edge of 3 pixels and B-factor of -137). Program suites EMAN2 [42], Chimera [43, 44], Coot [45] and Pymol (Schroedinger, LLC) were also used for visualization, analyses and figure preparation throughout the entire process. Rigid-body fitting of coordinates from PDB entry 4GQB to the reconstructed map was accomplished using Chimera [43, 44] and Coot [45].

## Results

**Fig 2:**
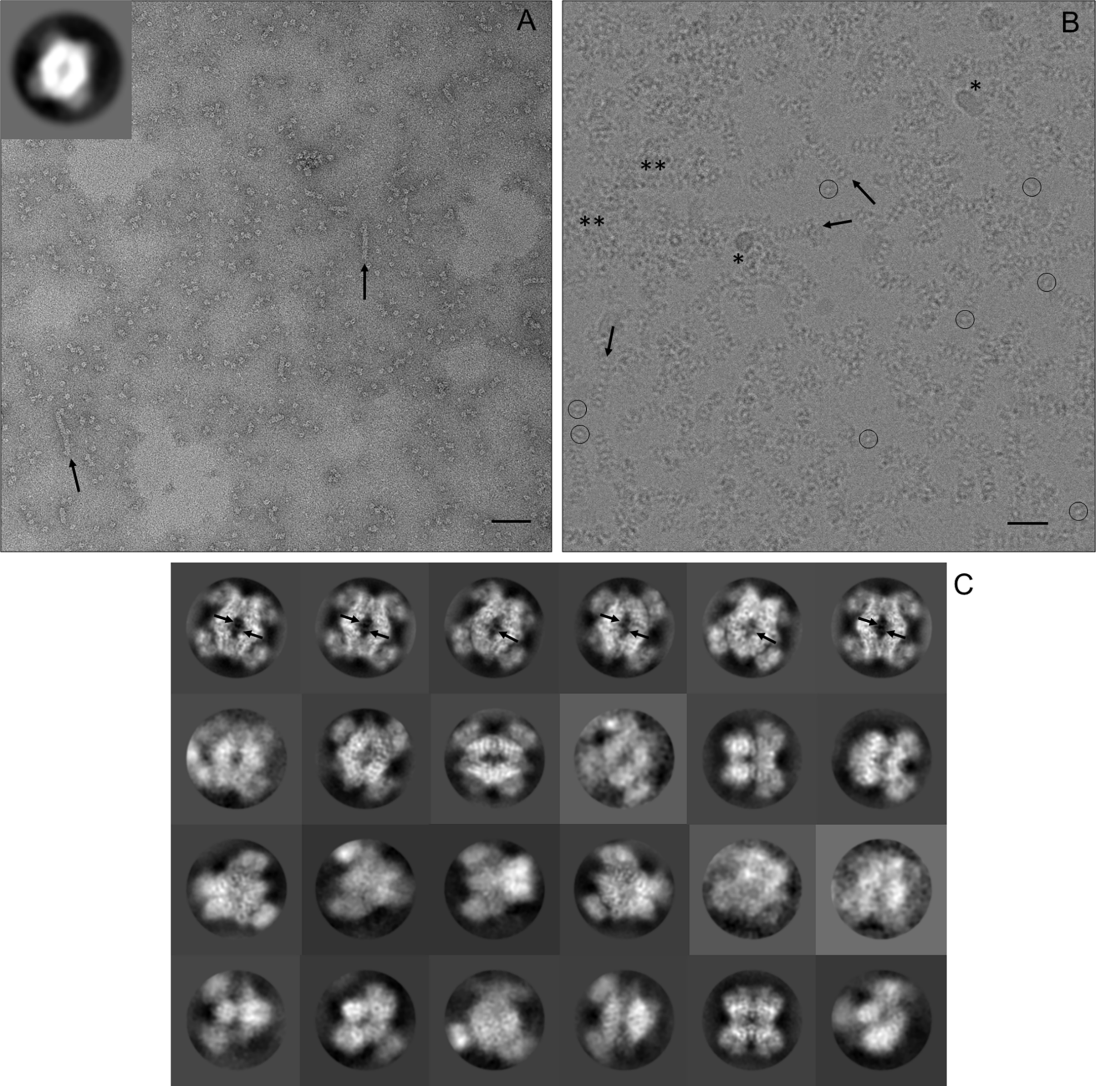
Raw micrographs and 2D class averages. (A) A section of a negative stained grid imaged with an actual magnification of 50,607x is shown. The inset in the top left corner shows the 2D class average corresponding to over 50% of the observed particles. The diamond-like shape of the PRMT5 core is well-resolved, but the MEP50 is poorly resolved. The bottom right scale bar is 90 nm. (B) A section of PRMT5:MEP50 particles embedded in vitreous ice is shown. The image is a motion corrected summation of 40 individual movie frames at an actual magnification of 50,000x. A low contrast, central ‘donut hole’ is visible in individual particles (circled). Particles of similar orientation to the (A) inset appear like ‘headless turtles’. Ice and/or ethane contamination and aggregated protein are indicated by * and **, respectively. The bottom right scale bar is 30 nm. (C) Representative 2D class averages calculated from cryo-images are shown with inverted contrast. The maximal PRMT5:MEP50 particle dimension is approximately 15 nm. Black arrows highlight small filamentous aggregates in (A) and (B), and pore loop density for residues 488–493 in (C).

Negative stain imaging (Fig 2A) was initially used to determine suitability of the PRMT5:MEP50 sample for EM analyses, to optimize buffer components and to estimate the protein concentration required to yield good particle densities in cryo-EM grids. Grids obtained with NaCl concentrations of 150 mM yielded images showing dispersed particles of ~15 nm and having an apparent ‘donut hole’ at the center of the particles (Fig 2A inset). Inclusion of low mM concentrations of the SAM competitive inhibitor, dehydrosinefungin [16, 26], was associated with increased particle density and reduced aggregation in negative stained grids, so the inhibitor was included as a buffer component for cryo-EM grid preparation. Protein concentrations of 0.23–0.58 mg/mL worked well for negative staining. Aggregation was significantly more prevalent in cryo-grids that were screened, even at lower dilutions. A grid prepared at a protein concentration of 0.38 mg/mL was chosen for high resolution cryo-imaging. Some small filamentous aggregates of ~15 nm width are observed at a low frequency, with or without dehydrosinefungin, in most negative stained and cryo- images (Fig 2A and 2B), indicating a slight propensity for the complex to associate in a side to side manner under these buffer conditions. However, it is unclear if this association is of any biologic relevance. The cryo-electron micrographs appear sub-optimal in having crowded particle densities, significant amounts of protein aggregation and some ice and/or ethane contamination (Fig 2B).

Viewed along the dyad axis to its widest orientation, PRMT5 forms a diamond shaped core (Fig 2) decorated along each side with a MEP50 subunit. This projection predominates in images from negative stained grids (Fig 2A inset), representing a preferred orientation on carbon that accounts for over 50% of particle orientations. This view of the complex is also apparent in raw motion corrected cryo-EM images as ‘headless turtle’ particles having a low contrast ‘donut hole’ center (Fig 2B) and in 2D class averages of the particles in ice (Fig 2C). 2D class averaging was used to identify and remove groups of particles containing contaminating ice or ethane, bad pixels, aggregation, etc. Some 2D class averages (Fig 2C) showed clear structural details, such as loop density protruding into the central ‘donut hole’.

**Fig 3:**
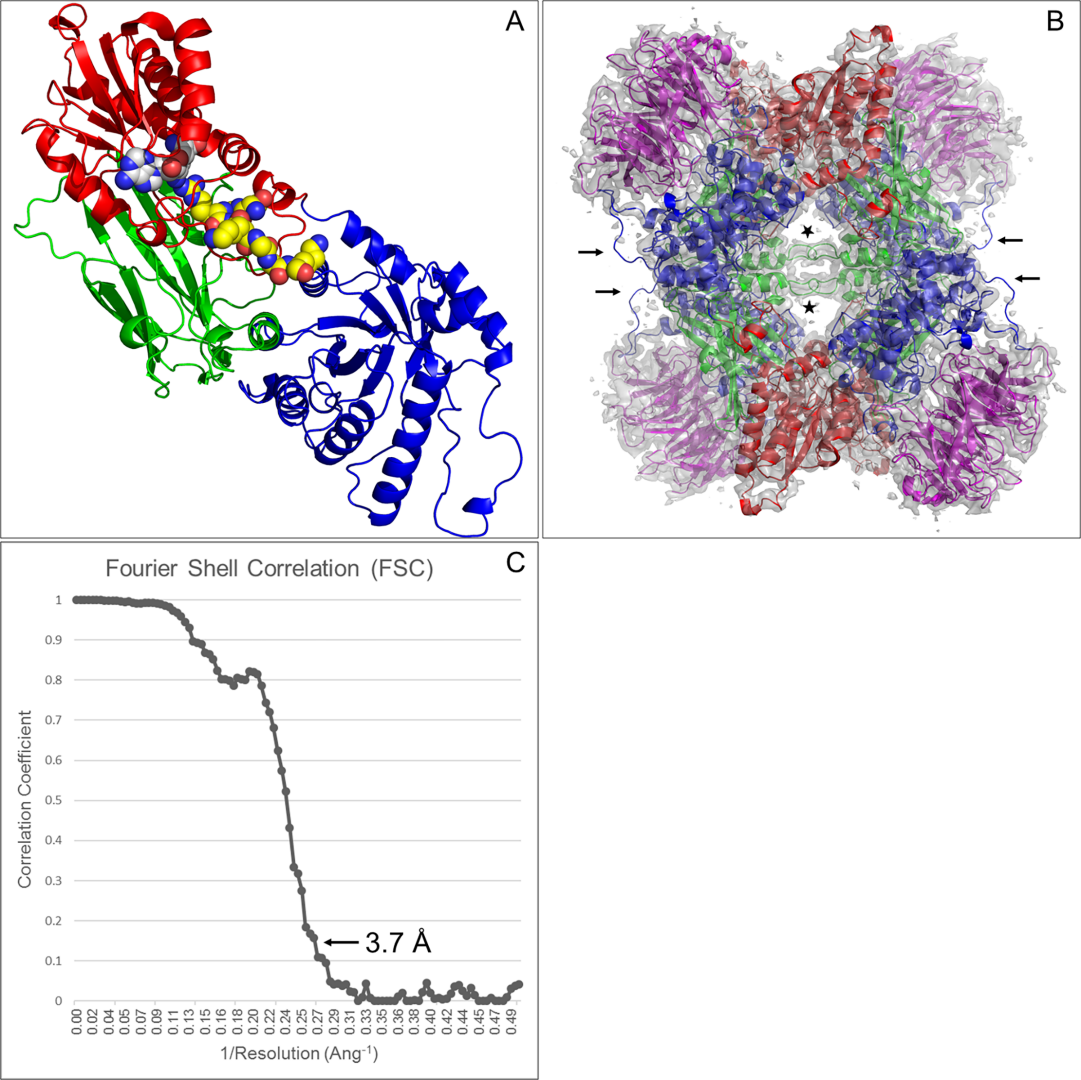
Structures of PRMT5 and the PRMT5:MEP50 complex. (A) An individual PRMT5 subunit from Protein Databank Entry 4GQB is represented as a ribbon diagram with dehydrosinefungin (white C atoms) and a histone H4 derived peptide (yellow C atoms) depicted as CPK spheres. The TIM barrel domain, Rossmann fold and β-barrel domains are colored blue, red and green, respectively. (B) The 3D reconstruction of the PRMT5:MEP50 complex, viewed along a dyad axis, is represented as a transparent gray map contoured at 3 rmsd (0.036). The PRMT5 subunits (colored as in Fig 3A) and the MEP50 subunits (colored magenta) were rigid body fit within the map as heterodimeric units. Black arrows highlight weak or missing density for PRMT5 residues 173–179. Density for loop residues 488–493 is highlighted by stars. Views of the 3D reconstruction along the other two central dyad axes are available in S3 Fig. (C) A resolution of 3.7 Å for the reconstruction is indicated at FSC = 0.143.

The PRMT5:MEP50 reconstruction is in very close agreement with the published 2.1 Å crystal structure [16] of the complex (Fig 3A and 3B). The human PRMT5:MEP50 complex structure has been well-characterized by X-Ray crystallography, with current PDB entries having resolutions ranging from 2.1 Å to 2.9 Å [16, 21, 22, 46], and a *Xenopus laevis* structure has been reported at 3.0 Å [17]. The human structures have all been solved in the same space group with a single PRMT5:MEP50 heterodimer present in the asymmetric unit, and all contain SAM or a SAM analog (S-adenosyl homocysteine, methylthioadenosine, sinefungin, dehydrosinefungin) bound in the active site. Individual PRMT5 subunits contain an N-terminal TIM barrel domain through residue 292 (Fig 3A), which serves as the exclusive binding partner of MEP50, and a methyltransferase domain (residues 293–637) which is composed of a Rossmann fold (residues 293–466) and a C-terminal β-barrel (residues 467–637). MEP50 is comprised of WD40 (Trp/Asp) repeats and a WE motif, which form a 7-bladed β-propeller structure (Fig 3B). The D2 symmetric heterooctameric biological assembly generated by applying crystallographic symmetry operations to the unique heterodimeric structure superimposes with that generated by rigid body fitting of the crystallographic heterodimer within the cryo-EM map (Fig 3B) with a rmsd of 0.3 Å. The core D2 symmetry of the complex is a result of extensive inter-subunit PRMT5:PRMT5 interactions involving both the N-terminal and catalytic domains [16], which give rise to a dimer of head to tail dimers association. The biological relevance of this human quaternary structure has been reviewed [12] and is supported by gel filtration, analytical ultracentrifugation measurements, and enzymatic activity analyses [3, 16].

**Fig 4:**
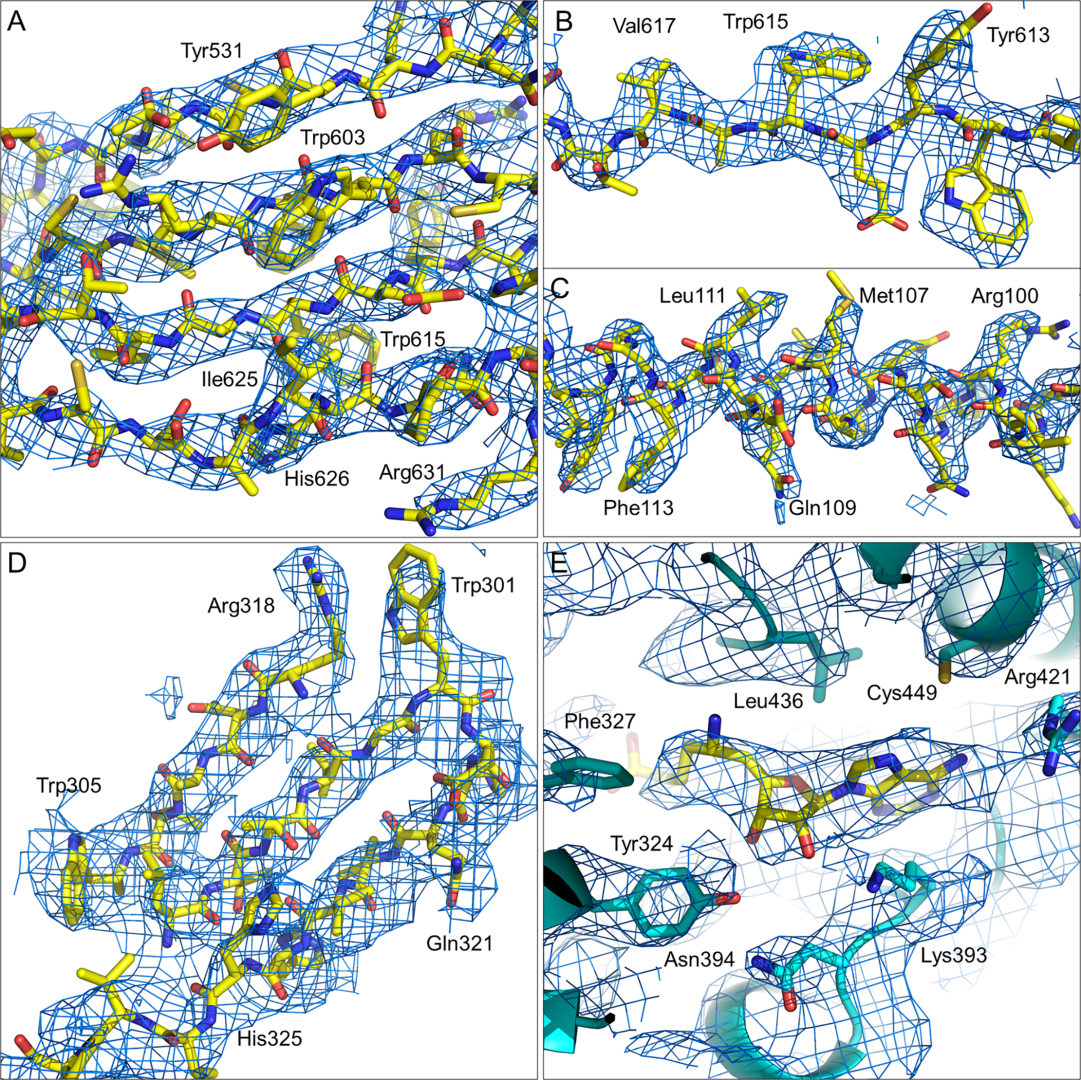
Details of the cryo-EM map in blue mesh. (A) A section of β-sheet present in the PRMT5 subunit shows good strand separation and side chain densities (B). (C) A section of α-helix in PRMT5 is shown with good helical pitch and bulky side chain density. (D) The seventh β-propeller in MEP50 is shown with good strand separation and bulky side chain density. (E) A peak in the Cryo-EM map, corresponding with binding of the dehydrosinefungin inhibitor (yellow C atoms), is well-resolved from the surrounding PRMT5 groups (cyan C atoms and ribbon). Carbon, nitrogen and oxygen atoms are colored yellow, blue and red, respectively, excepting where noted for panel E. The map is contoured at 4 rmsd (0.048) in panels (A), (B) and (C), and at 3 rmsd (0.036) in panels (D) and (E).

The overall estimate of 3.7 Å resolution (Fig 3C) for the reconstruction, based on fourier shell correlation (FSC = 0.143), is in good agreement with the level of molecular details apparent within the map. Fig 4 exemplifies some of the well-defined features, such as clearly resolved strands within β-sheet tertiary structures (Fig 4A) and side chain densities (Fig 4A–4E). It is also worth noting that it is not just the bulkiest aromatic sidechains that are resolved within the structure, but also many of the smaller side chains and side chains with higher numbers of rotatable bonds have observable density at least through the β- or γ- substituents. The peptide directionality is also apparent in many of the α-helices, such as that shown in Fig 4C. A greater degree of disorder occurs within the MEP50 domain at the periphery of the complex, but most features are still nicely defined, as in Fig 4D. The weakest region of density (arrows in Fig 3B) corresponds with where PRMT5 residues Thr173-Tyr179 have been modelled [16] in an extended loop that connects a MEP50 binding site (Arg164-Asn170) with a helix between Ser180-Asp197. This loop also shows strong signs of disorder within the crystal structure, with B-factors in the 55–110 Å^2^ range.

A final noteworthy feature of the cryo-EM map is the quality of density for the SAM competitive dehydrosinefungin inhibitor (Fig 4E). Dehydrosinefungin binding within the Rossmann fold domain has been described [16], with the nucleotide base amine group donating a H-bond to the Asp417 carboxyl group and the edge of the adenine ring accepting a H-bond from the Met420 peptide NH group [16]. The reconstructed density would independently allow a nearly identical fit of the inhibitor within the active site. The peak height and breadth of the cryo-EM map is sufficient to distinguish the adenine and ribose rings, as well as resolving the length of the amino acid group from the primary amine group. The quality of density is again consistent with the crystallographic thermal parameter gradient. Here the amino acid group (B-factors of 52–55 Å^2^) has a nearly negligible peak height within the cryo-EM map; the ribose ring is of intermediate map peak height and intermediate crystallographic B-factors, and the adenine group shows the highest map intensity and lowest B-factors (32–34 Å^2^). Other factors contributing to the weak density for the carboxylic acid group could include rotatable bond flexibility, a negative charge likely to be present at pH 7.4 and/or the absence of the histone H4 peptide in the sample used for cryo-EM imaging.

## Discussion

Recent advances in the field of biological cryo-EM have led to much well-founded enthusiasm for this next revolution in structural biology. Our biological understanding of molecular mechanisms and structures for entities ranging from the atomic to sub-cellular sizes has been and will continue to be impacted in a dramatic manner by this high resolution imaging technology. However, the application of cryo-EM in published drug discovery or structure-based drug design reports remains largely absent. At the time of this paper’s preparation, there are fewer than 100 SP cryo-EM maps present in the EMDB for human samples at a sufficient resolution (< 4 Å) to resolve low molecular weight ligands from surrounding protein groups within a binding site. Also, while there are notable examples of small ligands resolved within cryo-EM structures [28–31, 47], they represent a very small fraction of existing high resolution maps. There also remains a notable absence of high resolution cryo-EM structures for therapeutic proteins, including monoclonal antibodies (mAbs) or antigen binding fragments (Fabs) derived from therapeutic mAbs.

The results presented here represent a first published example of a SP cryo-EM reconstruction of the PRMT5:MEP50 complex and provides an additional ‘proof of concept’ among a growing body of work demonstrating the feasibility and utility of cryo-EM structure determination for proteins of high interest in human biology and biomedical research. Despite a fairly modest overall resolution, relative to crystal structures for this target, the 3.7 Å map appears qualitatively sufficient to assign a majority of the amino acid sequence within the structure. Furthermore, the level of detail present within the map is sufficient for identifying both the binding site and the binding orientation of a small molecule inhibitor having a molecular weight of only 379.4 Da. This ability to experimentally determine binding orientation is foundational to the use of structural information in structure-based drug design, and demonstrates that small molecule ligand orientations can be observed even in fairly modest quality cryo-EM maps. Additionally, with a number of other binding partners known to be of importance in PRMT5 function, this work provides a strong foundation for the future use of cryo-EM technology in the study of PRMT5 interactions with adapter proteins like pICln and CoPR, and substrates such as histone assemblies within nucleosomes.

Finally, the overall map quality described above is particularly remarkable, given the suboptimal appearance of the raw cryo-electron micrographs used in the 3D reconstruction. Micrographs containing close-packed virus particles have previously been used in high resolution 3D reconstructions [48], with an advantage noted in improving data collection throughput by increasing the number of particles per image. The PRMT5:MEP50 results presented here suggest that routine approaches to cryo-EM data processing can overcome significant sample quality issues of particle crowding and aggregation to yield 3D reconstructions of good overall quality and interpretability. The ability to routinely integrate real time data processing and analysis in parallel with data collection will be of critical importance in the future to identifying samples of apparently low visual quality that can nevertheless yield useful final results.

## Supporting information

S1 Fig PRMT5:MEP50 purification. (A) A SDS-PAGE gel is shown for the PRMT5:MEP50 sample used in the cryo-EM studies described here. Lanes 1, 2, 3 and 4 correspond to molecular weight markers, cellular lysate, FLAG column flow through and FLAG column eluate, respectively. (B) The preparative gel filtration chromatogram corresponding to the final purification step is shown (blue trace corresponds to absorbance units at 280 nm). Fractions were pooled to eliminate high molecular weight shoulder components. No low molecular weight peak fractions were observed.
(TIF)

S2 Fig Raw cryo-electron micrographs and auto-picked particles. Two example electron micrographs, low-pass filtered at 20 Å, are shown at a magnification of 50,000x. The electron micrograph in (A) was acquired with a defocus value calculated at 4.1 μm and corresponds with that shown in Fig 2B. The 164 Å diameter green circles in (B) indicate the positions of 593 auto-picked particles from (A). The electron micrograph in (C) was acquired with a defocus value calculated at 2.3 μm. The 164 Å diameter green circles in (D) indicate the positions of 484 auto-picked particles from (C). The number of auto-picked particles for the 193 cryo-images used in the 3D reconstruction range from 484 to 679.
(TIF)

S3 Fig Views of the PRMT5:MEP50 3D reconstruction and model. The map is shown as gray surface in (A) and (B). (A) Black arrows indicate positions of side chain density apparent for PRMT5 residue Tyr286. This view corresponds to a 90° rotation about the Y-axis of the view shown in Fig 3B. The view in (B) corresponds to a 90° rotation about the X-axis in (A), followed by a 90° rotation about the resulting Z-axis. The ribbon diagram is colored as described for Fig 3. (C) Density apparent within the active site and corresponding to the bound dehydrosinefungin inhibitor is shown in blue mesh. The view is related to that shown in Fig 4E by an approximate rotation of 90° about the X-axis. The position and conformation of the inhibitor model shown is the result of rigid body fitting the 4GQB.pdb coordinate file to the 3D reconstructed map. The map is contoured at a level of 3.0 rmsd (0.036) in (A), (B) and (C).
(TIF)
