# Supplementary figures and images for: Cryo-electron microscopy structure of a human PRMT5:MEP50 complex
Source: PLoS One. 2018 Mar 8;13(3):e0193205. doi: 10.1371/journal.pone.0193205 (PMC5843215; doi:10.1371/journal.pone.0193205)

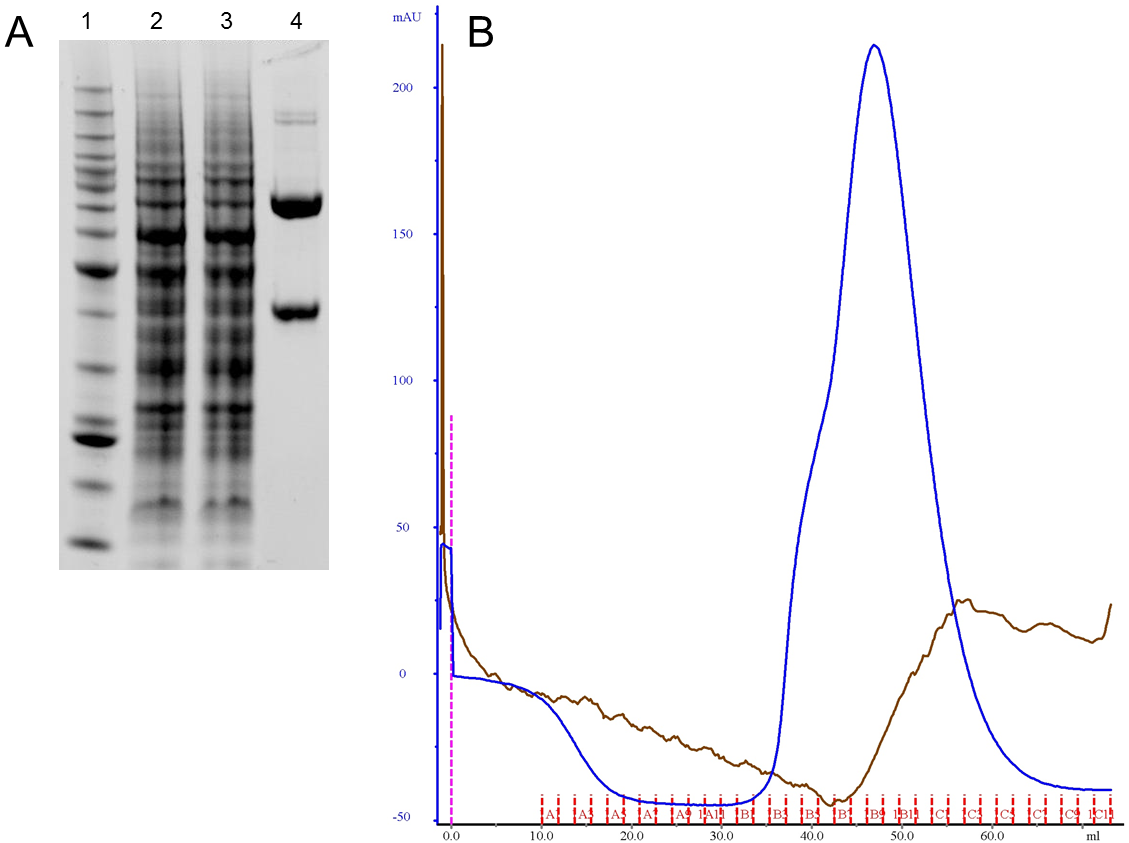

Supplement: S1 Fig — (A) A SDS-PAGE gel is shown for the PRMT5:MEP50 sample used in the cryo-EM studies described here. Lanes 1, 2, 3 and 4 correspond to molecular weight markers, cellular lysate, FLAG column flow through and FLAG column eluate, respectively. (B) The preparative gel filtration chromatogram corresponding to the final purification step is shown (blue trace corresponds to absorbance units at 280 nm). Fractions were pooled to eliminate high molecular weight shoulder components. No low molecular weight peak fractions were observed. (TIF) [file pone.0193205.s001.tif]

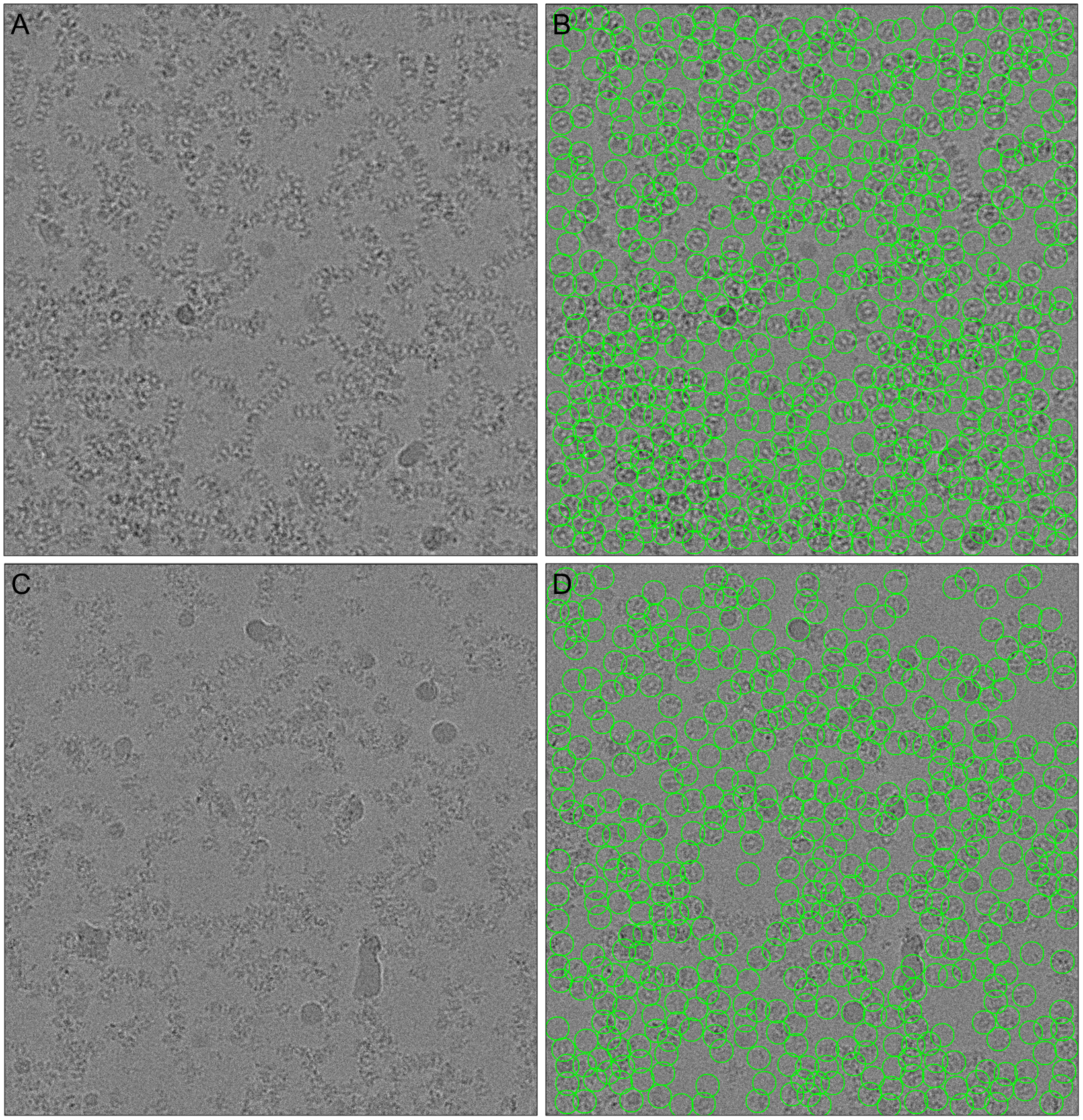

Supplement: S2 Fig — Two example electron micrographs, low-pass filtered at 20 Å, are shown at a magnification of 50,000x. The electron micrograph in (A) was acquired with a defocus value calculated at 4.1 μm and corresponds with that shown in Fig 2B. The 164 Å diameter green circles in (B) indicate the positions of 593 auto-picked particles from (A). The electron micrograph in (C) was acquired with a defocus value calculated at 2.3 μm. The 164 Å diameter green circles in (D) indicate the positions of 484 auto-picked particles from (C). The number of auto-picked particles for the 193 cryo-images used in the 3D reconstruction range from 484 to 679. (TIF) [file pone.0193205.s002.tif]

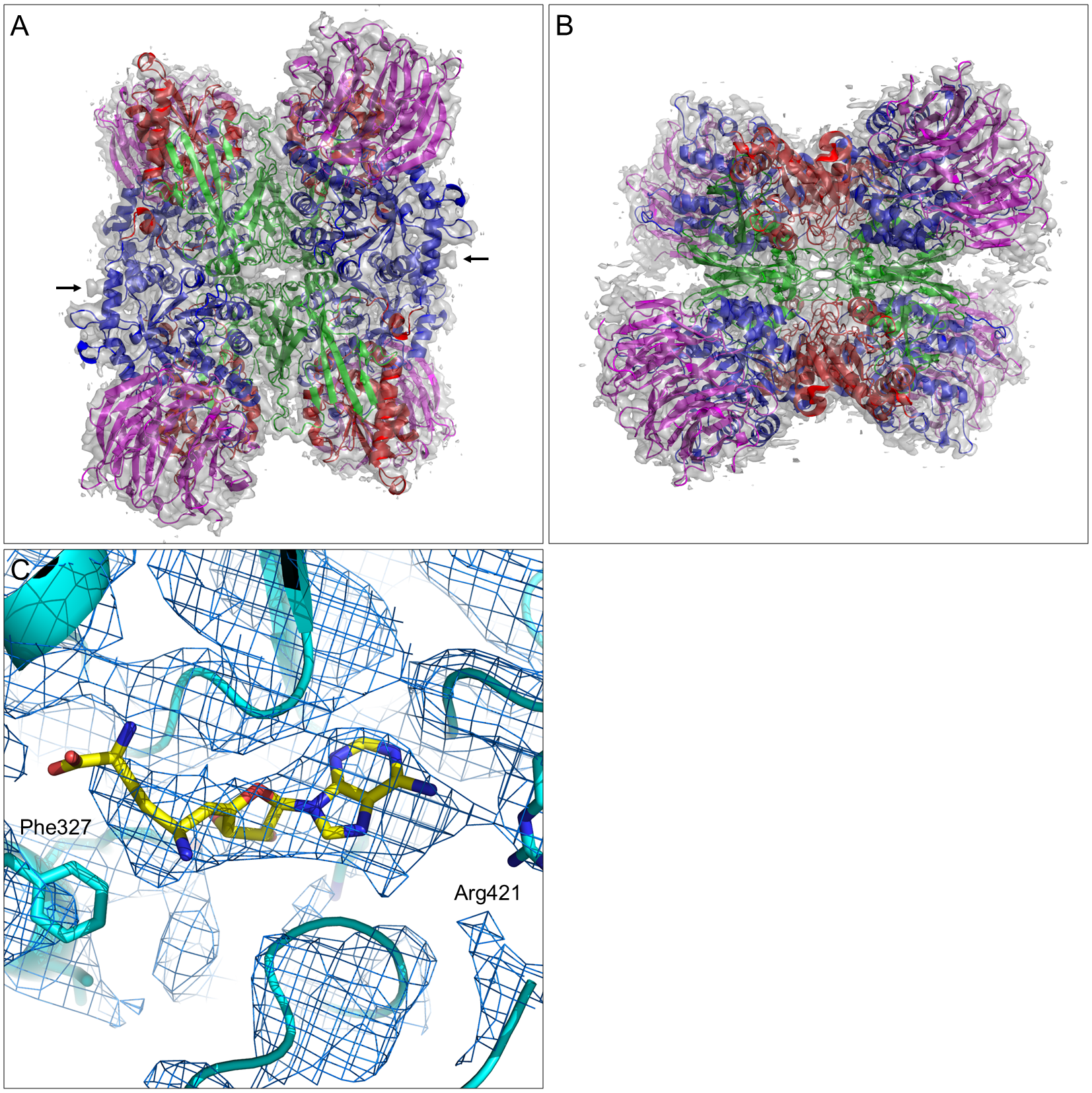

Supplement: S3 Fig — The map is shown as gray surface in (A) and (B). (A) Black arrows indicate positions of side chain density apparent for PRMT5 residue Tyr286. This view corresponds to a 90° rotation about the Y-axis of the view shown in Fig 3B. The view in (B) corresponds to a 90° rotation about the X-axis in (A), followed by a 90° rotation about the resulting Z-axis. The ribbon diagram is colored as described for Fig 3. (C) Density apparent within the active site and corresponding to the bound dehydrosinefungin inhibitor is shown in blue mesh. The view is related to that shown in Fig 4E by an approximate rotation of 90° about the X-axis. The position and conformation of the inhibitor model shown is the result of rigid body fitting the 4GQB.pdb coordinate file to the 3D reconstructed map. The map is contoured at a level of 3.0 rmsd (0.036) in (A), (B) and (C). (TIF) [file pone.0193205.s003.tif]
